# Developing and evaluating a Disaster Management Assessment Tool for Health Care Practitioners

**DOI:** 10.1186/s12873-025-01199-8

**Published:** 2025-03-06

**Authors:** Sara Elshami, Mohamed Izham Mohamed Ibrahim, Manar E. Abdel-Rahman, Hanan Abdul Rahim, Banan Mukhalalati

**Affiliations:** 1 Department of Clinical Pharmacy and Practice, College of Pharmacy, QU Health, Qatar University, PO Box 2713, Doha, Qatar; 2 Public Health Department, College of Health Sciences, QU Health, Qatar University, PO Box 2713, Doha, Qatar; 3 Clinical Pharmacy and Practice Department, Director of Integrated Learning, Vice President for Medical and Health Sciences Office, Qatar University, PO Box 2713, Doha, Qatar

**Keywords:** Disasters management, Healthcare practitioners, Assessment tools, Validity, Reliability

## Abstract

**Background:**

Over the last fifty years, the frequency and intensity of disasters have escalated, highlighting the importance of healthcare practitioners (HCPs) being thoroughly prepared for disaster management. Despite this pressing need, there is a notable lack of well-developed and rigorously evaluated assessment tools to evaluate disaster preparedness among HCPs across various disciplines and disaster scenarios. This study aims to develop and evaluate a Disaster Management Assessment Tool for Health Care Practitioners (DMAT_HCP).

**Methods:**

The DMAT_HCP was designed following the four stages of the Disaster Management Framework and a literature review of similar previously validated tools. Content validity was assessed through two rounds of review by nine and six experts, whereas face validity was assessed by 11 HCPs. DMAT_HCP was tested on 107 HCPs from different health disciplines and settings to evaluate the structural (factor analysis) and construct (convergent and divergent) validities as well as internal consistency reliability.

**Results:**

DMAT_HCP comprised five Likert scales that assess the preparedness and readiness of HCPs for disaster, with satisfactory content validity indices (CVI > 0.83 for six experts). Factor analysis of the entire set of DMAT_HCP items suggested six factors: knowledge, two sub-domains of attitude, practice, willingness to practice, and organization-based management, which together accounted for 77.9% of the variance in the data. Convergent and divergent validity analyses showed that all items within a section had a correlation coefficient greater than 0.4 with their corresponding section score, and they were more strongly correlated with their own section than with scores from other sections. Cronbach’s alpha values for the individual sections ranged from 0.89 (attitude) to 0.97 (organization-based management), and the overall Cronbach’s alpha for the DMAT_HCP was 0.90.

**Conclusions:**

This study substantiated that DMAT_HCP is both conceptually and methodologically valid and reliable. It has demonstrated strong content validity, accurately measures the intended constructs, and effectively distinguishes between unrelated constructs. The tool also exhibited excellent internal consistency reliability across its components. The tool offers a comprehensive, globally applicable assessment of disaster management, suitable for use across various healthcare professions, settings, disaster contexts, and management phases.

## Background

Disasters are defined as “serious disruptions to the functioning of a community that exceed its capacity to cope using its own resources” [1]. Over the past five decades, the frequency and severity of disasters have increased, impacting countries worldwide and posing significant challenges to global public health and healthcare systems [2, 3]. Effective disaster management has become critical in mitigating these challenges and protecting communities from the adverse consequences of such events [4, 5].

Disaster management refers to “the organization, planning, and application of measures preparing for, responding to, and recovering from disasters” [6]. It is underpinned by the Disaster Management Framework, a systematic and well-established approach that outlines the cycle of four interconnected stages: mitigation, preparedness, response, and recovery [7, 8]. Mitigation focuses on minimizing disaster impact, preparedness emphasizes training and resource allocation, response involves immediate interventions, and recovery aims to restore functionality and rebuild infrastructure [7, 8]. Despite variations in its implementation across countries, the framework provides a universal strategy to reduce suffering, alleviate the consequences of disasters, and support community resilience [7].

Healthcare practitioners (HCPs), across various healthcare disciplines, including physicians, nurses, paramedics, pharmacists, and allied health professionals, play an indispensable role in disaster management, ensuring that the necessary interventions are carried out effectively and efficiently. Their roles and responsibilities are directly linked to each stage of the disaster management cycle, which extend beyond routine care, requiring risk assessment, timely decision-making, adaptability under resource-limited conditions, and implementing public health interventions [4, 5, 9]. During the mitigation phase, HCPs identify vulnerabilities, promote preventive measures like vaccinations, and provide public health education to reduce the potential impact of disasters. In the preparedness phase, they conduct training and raise awareness to enhance emergency readiness across communities and healthcare systems. In the response phase, they provide critical care, manage casualties, and coordinate interventions to reduce mortality and morbidity, ensuring that healthcare delivery continues under emergency conditions. Lastly, in the recovery phase, HCPs address long-term health impacts, support rehabilitation efforts, and help rebuild healthcare infrastructure, restoring functionality and resilience to the community [4, 5, 9]. To fulfill these responsibilities, HCPs require adequate knowledge, technical skills, and a proactive attitude, all of which can be achieved through targeted education and training [10, 11]. Their preparedness to act promptly and confidently in disaster scenarios underpins the success of disaster management efforts [9].

The global emphasis on disaster management has triggered initiatives that aim to better prepare HCPs for disasters. Several assessment instruments have been developed to evaluate a variety of dimensions, including the preparedness competencies of HCPs [12]. The preparedness competencies of HCPs have been assessed by evaluating their knowledge, skills, attitudes, confidence, and willingness to act effectively during disasters [13, 14]. Some instruments that were developed evaluated the preparedness of HCPs for disasters in a general context [12, 15–17], while others focused on specific disaster types [18–21]. Furthermore, certain studies have examined preparedness and readiness for practice during disasters across multiple healthcare professions [17, 22], whereas others have concentrated on specific healthcare professions, such as emergency medical services, nursing, medicine, or pharmacy [12, 23–28]. For example, in the United States, the Emergency Preparedness Information Questionnaire (EPIQ) is employed to assess general disaster knowledge across multiple phases, such as prevention, mitigation, response, and recovery [29]. Despite that, its applicability may be limited due to its exclusive focus on nursing professionals and the absence of a robust theoretical framework, potentially hindering its generalizability across broader HCP populations [29]. Likewise, in Brazil, the Nurses’ Disaster Response Competencies Assessment Questionnaire (NDRCAQ) evaluates nursing competencies in disaster response, but its exclusive focus on nursing professionals limits its comprehensiveness across all healthcare roles and disaster management phases [28]. The Disaster Preparedness Evaluation Tool (DPET) evaluates preparedness specifically for biological disasters by focusing on disaster knowledge, skills, and personal preparedness [3]. Although it offers a comprehensive assessment of biological disaster preparedness, it remains confined to a single disaster type, restricting its relevance to other disaster scenarios [3]. Similarly, the Provider Response to Emergency Pandemic (PREP) tool, designed for multi-profession preparedness, predominantly addresses responses to biological emergencies, placing less emphasis on practical competencies and crucial disaster management phases such as recovery and mitigation [21]. In Ireland, the Major Emergency Preparedness in Ireland Survey (MEPie) measures knowledge related to major emergency planning and core clinical response activities [17]. However, it largely emphasizes theoretical knowledge and self-assessed competence which may potentially limit its ability to fully evaluate practical, real-world preparedness skills [17].

Within the Arab region, the Disaster Nursing Core Competencies Scale (DNCCS) was developed by Al-Thobaity et al. [12] in Saudi Arabia to assess core competencies in nursing for disasters in general and across various phases, including knowledge, roles, and barriers [12]. However, it is limited to nursing professionals, restricting its broader applicability across healthcare professions [12]. In contrast, Nofal et al. [11, 30] developed tools that assess preparedness among HCPs from multiple professions in Saudi Arabia [11, 30]. While these tools expand the scope by including different healthcare roles, they have limited validity evidence, such as content validity, and primarily focus on bioterrorism preparedness [11, 30]. In Jordan, the tool developed by Alwidyan et al. [23] focuses on emergency medical services (EMS) providers and primarily addresses pandemic-related disasters, assessing attitudes and concerns about working during disease outbreaks [23]. While this tool offers valuable insights into pandemic preparedness, its scope is limited to a single disaster type and does not assess other disaster types or the complete range of disaster management phases [23]. Similarly, in Yemen, the tool developed by Al-Hunaishi et al. [31] assesses HCPs’ willingness to engage in disaster management and their self-efficacy across both biological and natural disasters [31]. While it covers a range of disaster types, the psychometric validation is limited, which may affect its reliability and generalizability [31].

While these tools make valuable contributions to the field within their respective contexts, there are still opportunities for enhancement. Despite the acknowledged importance of disaster management and healthcare provider (HCP) preparedness, a significant gap exists in the availability of robust, well-developed, and thoroughly validated assessment tools that are inclusive and adaptable. Such tools should be capable of assessing disaster management across all healthcare professions, including physicians, paramedics, nurses, pharmacists, and allied health professionals, and be applicable to a wide range of disaster scenarios. Additionally, there is a clear need for a comprehensive tool that covers all phases of disaster management. This is crucial for effective disaster response planning and ensuring that HCPs are adequately prepared to manage and respond to the complexities of diverse disaster situations.

The aim of this study is to develop and evaluate the Disaster Management Assessment Tool for Health Care Practitioners (DMAT_HCP), a comprehensive and versatile tool designed to offer a multi-profession, multi-context approach to assessing HCPs’ preparedness across all disaster management phases in healthcare settings globally.

## Methods

A methodological study for tool development and evaluation of the psychometric properties of the Disaster Management Assessment Tool for Health Care Practitioners (DMAT_HCP) was employed [32]. The study was conducted in two phases: 1) tool development and 2) tool evaluation. The DMAT_HCP was developed using a deductive items pool generation strategy, where items were generated from the literature review and the existing assessment tools. These items were systematically mapped to the four stages of the Disaster Management Framework. Content validity was conducted to ensure that the tool comprehensively represented the domain of disaster management across diverse healthcare settings, disciplines, and disaster contexts. Drawing from best practices in scale development [32], content validity involved a systematic review by subject-matter experts in healthcare, public health disaster preparedness, and survey development and evaluation to assess the relevance, technical quality, and breadth of the items. Additionally, feedback from target population representatives (11 HCPs from diverse disciplines, including nursing, medicine, paramedicine, pharmacy, dentistry, laboratory technology, and other allied health fields in Qatar) ensured the clarity and appropriateness of the items. These steps were essential to establish that the DMAT_HCP captured the full scope of disaster management without omitting critical components while ensuring its practical relevance and usability.

The evaluation phase employed a cross-sectional design involving 107 HCPs from various clinical disciplines and healthcare settings in Qatar, including Hamad Medical Corporation (HMC), Primary Health Care Corporation (PHCC), and the Ministry of Public Health (MoPH). The DMAT_HCP was administered as a 50-item online self-administered questionnaire developed during the first phase. The evaluation of DMAT_HCP aimed to provide statistical evidence of its theoretical alignment and measurement accuracy. DMAT_HCP was evaluated for structural validity through factor analysis to identify whether the items grouped into factors that represent the theoretical dimensions. Construct validation (convergent and divergent validities) was also conducted in the evaluation phase to ensure that the DMAT_HCP measured the intended constructs of disaster management accurately and each construct was conceptually distinct from other unrelated or overlapping constructs. Furthermore, an internal consistency analysis was conducted to establish the tool’s reliability.

Ethical approval to conduct the study was obtained from the Qatar University Institutional Review Board (QU-IRB) [approval number: QU-IRB 1759-EA/22], Hamad Medical Corporation (HMC-IRB) [approval number: MRC-03-22-392], and Primary Health Care Corporation (PHCC-IRB) [approval number: PHCC/DCR/2022/06/041].

### Phase 1: Tool development

#### Item generation

The objective of this phase of the study was to develop an assessment tool that assesses the perceptions of HCPs regarding their disaster management 1) knowledge, 2) attitude, 3) practices, 4) willingness to continue practicing duties during disasters, 5) as well as their perceptions of the level of preparedness to manage disasters among healthcare organizations.

The first section, *Knowledge*, assesses HCPs’ understanding and awareness of essential disaster management components. This includes their knowledge of reporting procedures, awareness campaigns, collaboration in mitigation, role clarity, access to information, organizational protocols, as well as national and organizational response systems and post-disaster roles. The second section, *Attitude*, evaluates HCPs’ beliefs regarding the importance of disaster plans at both organizational and national levels, their confidence in working independently, their interest in professional development, the role of media in disaster management, their readiness for rapid service escalation, and their recognition of the essential nature of their roles, along with their ability to implement plans and utilize technology during and after disasters. The third section, *Practice*, examines the extent to which HCPs engage in disaster management activities. It focuses on their participation in training, drills, and professional development programs, communication with authorities, prioritization of safety for themselves and others, volunteer management, and adherence to established disaster plans. The fourth section, *Willingness to Continue Practicing Duties During Disasters*, assesses HCPs’ resilience and commitment to fulfilling their responsibilities during and after disasters, particularly under challenging conditions such as inadequate training, mental preparedness, and the absence of necessary equipment and safety measures. The final section, *Organization-Based Disaster Management*, evaluates HCPs’ perceptions of their organization’s preparedness. This includes their assessment of the organization’s disaster plan, communication systems, surveillance mechanisms, availability of medical equipment, and the frequency and effectiveness of disaster drills.

A deductive items pool generation strategy was used, where items were generated from the literature review and the existing assessment instruments [33, 34]. The findings of a scoping review conducted by the research team (unpublished work) guided the development of the current assessment tool, by identifying the key instruments that have similar objectives with sound development and evaluation approaches [3, 11, 12, 16, 17, 21–23, 28, 29]. The research team sought permission from the original developers of these instruments to utilize (i.e., adopt or adapt) some of the items in the process of deductive items pool generation of the current assessment tool. The items of these instruments were mapped to the Disaster Management Framework [8]. Furthermore, new items were developed to align with the stages of the framework, as deemed relevant. The development phase involved consultation with an expert scholar in the field of disaster management and preparedness to improve the content and the structure of the DMAT_HCP, as well as with members of the research team.

#### Content validity

After the initial development of the DMAT_HCP, several cycles of revisions were conducted. Then, content validity, which refers to the “adequacy with which a measure assesses the domain of interest”, was conducted as outlined below [34]. DMAT_HCP was subjected to expert evaluation by a convivence sample of national and international experts. In the first round of evaluation, thirteen experts were selected based on their expertise in healthcare (n = 13), public health disaster preparedness (n = 8), and survey development and evaluation (n = 8). The selected experts were invited by email to share their critical evaluation of the clarity and relevance of the items to the duties of HCPs at times of disaster. The relevance of DMAT_HCP was assessed through a four-point Likert scale (i.e., 1: not relevant, 2: somewhat relevant, 3: quite relevant, and 4: very relevant) [35–39]. Similarly, the authors adapted the four-point Likert scale (i.e., 1: not clear, 2: somewhat clear, 3: quite clear, and 4: very clear) to assess the clarity of DMAT_HCP. Content validity was evaluated qualitatively by a review of the feedback received by the experts and quantitatively by determining the average content validity indices for relevance and clarity of the items (i-CVI) and scales (s-CVI) [35]. Items were removed if they did not pass the acceptable score of i-CVI for relevance, or modified if they did not pass the acceptable score of i-CVI for clarity [35]. The acceptable score of CVI values ranges from 0.78 to 1.00, according to the number of experts evaluating the assessment tool [35–39]. A CVI of at least 0.78 was considered acceptable if nine or more experts evaluated the assessment tool, 0.83 if six to eight experts, 1.00 if three to five experts, and 0.80 if two experts [35–37, 39]. Moreover, the experts were asked to add any additional questions to capture healthcare professionals’ perceptions of the four stages of disaster management.

In the second round of evaluation, a different convenience sample of six experts was selected and invited to conduct a more extensive review of the clarity of the items. After the second round of evaluation, the CVIs were recalculated, and no further rounds of evaluation were deemed necessary.

##### Target population evaluation

The modified version of DMAT_HCP, based on the expert evaluation, was shared with a convenience diverse sample of HCPs from the target population through SurveyMonkey® (Survey Monkey Inc., San Mateo, California, USA). Eleven HCPs were selected based on their health disciplines and were invited through email to review whether the items of the tool were appropriate for the targeted construct and assessment objectives.

### Phase 2: Tool evaluation through pilot testing

#### Setting

This phase employed a cross-sectional design involving HCPs from various healthcare settings in Qatar, including Hamad Medical Corporation (HMC), Primary Health Care Corporation (PHCC), and the Ministry of Public Health (MoPH).

In Qatar, the highest health authority is the MoPH, which is responsible for setting national healthcare priorities, regulating and overseeing healthcare systems, and offering services to suit those requirements [40]. The two key sectors of the healthcare system in Qatar (i.e., the private and the public) operate under the regulatory framework set by the MoPH [40]. Within the public healthcare sector, MoPH oversees organizations such as the HMC and the PHCC [40]. The HMC covers approximately 13 hospitals, including specialist and community hospitals. The PHCC manages around 31 centers, which are strategically situated in different areas around the country to ensure the accessibility of primary healthcare services to meet the needs of the population [40]. In the private sector, various private hospitals, including Al Emadi, Al-Ahli, Turkish, and Aster hospitals, as well as over 70 polyclinics, contribute to the comprehensive healthcare landscape [40].

#### Participants

The sampling frame in this study constituted all HCPs from different health disciplines (e.g., nurses, physicians, pharmacists, dentists, and allied health professionals) who work in the three different clinical settings (i.e., HMC, PHCC, and MoPH). The eligibility criteria included HCPs who were: 1) above 18 years old, 2) licensed as a physician, a nurse, or an allied healthcare professional (including paramedics, laboratory technologists, and physio- and respiratory therapists), 3) practicing in PHCC, HMC, or MoPH- Qatar, and 4) working in PHCC, HMC, or MoPH during the Gulf Crisis 2017 and/or COVID-19 disasters.

The inclusion of participants from diverse settings and disciplines was intended to facilitate a comprehensive evaluation of disaster management in Qatar and to examine the tool’s applicability across different healthcare levels and professional domains.

HCPs employed in the private sector were excluded from this study due to the predominance of public-sector healthcare services in Qatar. Moreover, anticipated logistical challenges associated with data collection and participant recruitment from private-sector organizations further informed this exclusion.

#### Sample size, and sampling

There were 31 primary healthcare centers and approximately 4,818 primary HCPs (i.e., 1010 physicians, 2182 nurses, 393 pharmacists, 226 dentists, and 1007 allied health professionals). In addition, there were 13 hospitals under HMC and approximately 21,157 secondary and tertiary HCPs (i.e., 3642 physicians, 11,281 nurses, 638 pharmacists, 164 dentists, and 5434 allied health professionals). Further, there were 15 health areas in the MoPH and approximately 61 HCPs (i.e., 12 physicians, 10 nurses, 1 dentist, and 38 allied health professionals). All eligible HCPs practicing at PHCC (n = 4487), HMC (n = 20395), or MoPH (n = 55) constituted the sampling frame in this study.

No consensus was established on the minimal required sample size for testing the psychometric properties of questionnaires (e.g., factor analysis), with the item-to-subject ratio ranging from 1:2 to 1:10 being one of the approaches [41–43]. The alternative approach specified the minimum sample size in absolute terms, with a recommendation of at least 100 participants [43–45]. In this study, the required sample size was determined based on a minimum recommended size (i.e., 1:2) of 100 HCPs for a 50-item questionnaire. However, 350 HCPs were approached to account for potential non-response. HCPs were selected for the evaluation of psychometric properties using a stratified sampling method based on healthcare professions/disciplines, as follows: allied health professionals: 86 (72 HMC, 1 MoPH, 13 PHCC), dentists: 5 (4 HMC, 1 PHCC), nurses: 184 (160 HMC, 24 PHCC), pharmacists: 14 (8 HMC, 6 PHCC), and physicians: 61 (47 HMC, 14 PHCC).

#### Data collection

The evaluation of psychometric properties of DMAT_HCP was conducted in a cross-sectional design. An email inviting the selected HCPs to participate in the study was sent to them. They were informed about the study purpose, the process of data collecting, the assurance of anonymity and confidentiality, and the voluntary nature of their involvement. A link to the self-administered DMAT_HCP questionnaire at SurveyMonkey® (Survey Monkey Inc., San Mateo, California, USA) with informed consent was included in the invitation emails. The survey was open for about three weeks between August and September 2023. To encourage participation, the HCPs received two reminder emails.

#### Validity and reliability analyses

The validity and reliability of DMAT_HCP were established through the assessments of the structural validity, construct validity (i.e., convergent and divergent validities), and internal consistency. Criterion validity and differentiation by known group evaluations were not conducted due to the lack of a gold standard questionnaire and the well-established knowledge regarding group-specific perceptions of disaster management [32]. All statistical analyses were performed using a standard software package STATA® version 18.1 (StataCorp LLC, Texas 77845 USA).

##### Structural validity: exploratory factor analysis (EFA)

EFA is known as a group of multivariate statistical techniques designed to uncover the smallest number of underlying constructs (also referred to as factors) that can reasonably account for and explain the observed correlation among a group of measured variables (also called observed variables) [46]. EFA is concerned with identifying and examining trends in the inter-item correlation (covariance) matrix which represents the loading of each observed variable on each factor [32]. EFA was initially employed in this study on the initial set of items in each section (12-item, 12-item, 10-item, 6-item, and 10-item scales, respectively) as a useful method to generate hypotheses on the structure of the data [47]. Measures of sampling adequacy for EFA, including the Kaiser–Meyer–Olkin (KMO > 0.5), significant Bartlett’s Test of Sphericity (BTS; p-value < 0.05), inter-item correlation coefficient (r > 0.3), and correlation determinant (>0.00001) were examined for each section separately [48], and the sample demonstrated appropriateness for factor analyses. Pett et al. [49] recommended the use of the principal components analysis (PCA) to develop preliminary solutions in EFA [49]. The eigenvalues (>1) and scree plots from PCA were examined to identify the number of components that capture meaningful variance in the data. This information guided the subsequent EFA by providing an initial estimate of the number of factors to extract [49, 50]. The factors were extracted using the iterated principal factor method as the data demonstrated a non-normal distribution and the retained factors from each scale were rotated using the varimax rotation. The varimax rotation method is an orthogonal rotation technique that reduces the number of variables with substantial loadings on each factor, which simplifies the interpretation of these factors [49, 51]. Total variance explained by the retained factors of 60% or greater was considered an acceptable target in the assessment of the relevance of the retained factors [52]. Items with high loading into one factor (>0.3) were retained; however, items with the highest loadings were the base for factor naming [32, 33, 53]. Items that demonstrated significant cross-loadings (>0.3) into two or more factors were subjected to theoretical and practical judgment, and were deleted if not explained by any of the factors [54]. In addition to conducting the analysis on separate sections of DMAT_HCP, an EFA was re-conducted on the entire set of DMAT_HCP items. This additional analysis aimed to further explore the overall structure of the questionnaire and evaluate it as a unified assessment tool for disaster management HCPs. The measures of sampling adequacy for EFA revealed a KMO value of 0.694, a significant Bartlett’s Test of Sphericity (BTS; *p*-value < 0.05), and a correlation determinant (>0.00001), yet, some inter-item correlation coefficients were < 0.3. Cooks distance was used to examine the outliers, as one of the most common statistics used to identify multivariate outliers. The analysis indicated the presence of 15 outliers, however, the outliers were retained to prevent a reduction in sample size if the outliers were removed, as this would render the analysis unfeasible.

##### Construct validity: convergent and divergent analyses

The construct validity of the DMAT_HCP is the theoretical relationship of the items to each other and the hypothesized sections. The construct validity was tested through the assessment of convergent and divergent validities [47]. Convergent validity was assessed by evaluating whether the items covering one section correlate with each other [33, 47]. A more stringent correlation (r > 0.4 or higher) was used to support convergent validity between the item and the overall sum score for the section to which it is supposed to belong [47]. Whereas the divergent validity assessment tested whether an item has a higher correlation with its hypothesized section than its correlation with the other sections [47]. The construct validity of the DMAT_HCP was tested twice; the first time after performing the EFA for each section of DMAT_HCP and the second time after performing the EFA for the entire set of items of DMAT_HCP. The ‘validscale’ command with the ‘tconvdiv(0.4)’ option in Stata was used to test for convergent and divergent validities [55].

##### Reliability testing

Reliability testing involves determining whether a scale or measurement produces repeatable and consistent findings [47]. Internal consistency was used in this study as a measure of internal reliability (i.e., the homogeneity and consistency of items in measuring the same concept) for each section of the DMAT_HCP using the item correlations [47]. Internal consistency was used to evaluate the extent to which the number of items in each section was adequate and that the items were interrelated through item-to-item correlations [47]. The internal consistency analysis was reported using Cronbach’s alpha, as one of the most predominantly employed statistics to examine scales reliability [33, 56]. Cronbach’s alpha was determined for each section of the DMAT_HCP as well as for the entire set of items of DMAT_HCP. Cronbach’s alpha of ≥ 0.7 was considered the minimum satisfactory value for acceptable internal consistency in this newly developed questionnaire [52].

## Results

### Phase 1: Tool development

#### Content validity

**Fig. 1:**
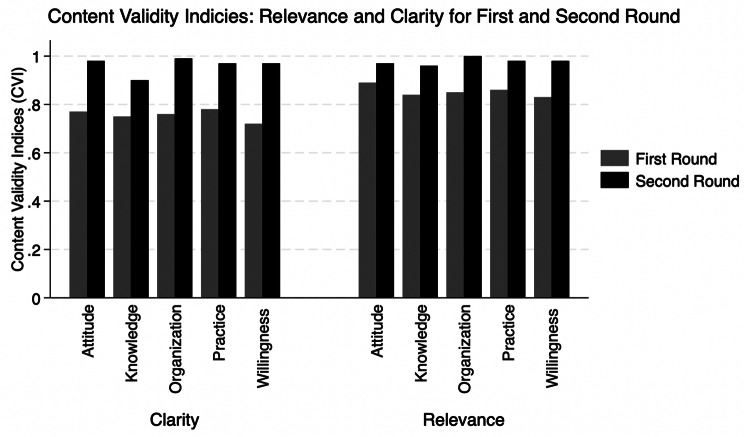
Content validity indices for the draft version of Disaster Management Assessment Tool for Health Care Practitioners (DMAT_HCP)

In the first round of the content validity evaluation, nine of the thirteen invited experts participated in the evaluation. Their expertise spanned across healthcare (n = 9), public health disaster preparedness (n = 5), and survey development and evaluation (n = 7). While all sections met the relevance criterion, not all of them satisfied the clarity criterion, necessitating a second round of evaluation. In the second round, the CVIs were recalculated based on feedback from six experts specializing in healthcare (n = 6), public health disaster preparedness (n = 4), and survey design and evaluation (n = 3). All sections were found to meet both the relevance and clarity criteria. Figure 1 provides the results of the CVI analysis for the two rounds of content validity evaluation.

##### Target population evaluation

The modified version of DMAT_HCP was then reviewed by a total of eleven HCPs (six males and five females) who participated in this phase, including one nurse, two physicians, two paramedics, two pharmacists, two laboratory technologists, and two physiotherapists. Most HCPs agreed on the relevance of the items to their disaster-related practices in Qatar, the appropriateness and sufficiency of the response options, the logical sequence in which the items were presented, and their willingness to respond to all questionnaire items without hesitation. Nevertheless, few participants highlighted the ambiguity of specific items, leading to further clarity enhancements. They also emphasized the necessity for a ‘not applicable’ option in certain sections, which was considered and incorporated into the tool. Of significant note, the questionnaire underwent additional shortening in response to frequent feedback from participants regarding the tool’s length.

#### DMAT_HCP: version for pilot testing evaluation

The expert and target population evaluations resulted in a version of DMAT_HCP comprising 50 items, in addition to 11 items about demographics and professional characteristics. The five key sections of the DMAT_HCP assessed the perceptions of HCPs of their disaster management in terms of A) knowledge, B) attitude, C) practice, D) willingness to continue practicing duties, and E) organization-based disaster management, as follows:

- Section A: Knowledge (12 items) evaluates HCPs’ understanding of disaster management concepts. Items in this section were rated using a 5-point Likert-type scale format (i.e., 1: no knowledge, 2: minimal knowledge, 3: basic knowledge, 4: adequate knowledge, or 5: superior knowledge).
- Section B: Attitude (12 items) assesses HCPs’ beliefs regarding various aspects of disaster management. Responses were rated using a 5-point Likert-type scale format (i.e., 1: strongly disagree, 2: disagree, 3: neutral, 4: agree, or 5: strongly agree).
- Section C: Practice (10 items) examines HCPs’ engagement in disaster management activities. This section employed a 5-point Likert-type scale format (i.e., 1: never, 2: rarely, 3: occasionally, 4: frequently, or 5: very frequently). This section also contained a nonordinal option of ‘not sure’.
- Section D: Willingness to Continue Practicing Duties During Disasters (6 items) measures HCPs’ readiness to continue performing their duties under disaster conditions. Items in this section were rated using a 5-point Likert-type scale format (i.e., 1: strongly unwilling, 2: unwilling, 3: neutral, 4: willing, or 5: strongly willing).
- Section E: Organization-Based Disaster Management (10 items) evaluates HCPs’ perceptions of their organization’s preparedness for disaster management. Responses were rated using a 5-point Likert-type scale format (i.e., 1: strongly disagree, 2: disagree, 3: neutral, 4: agree, or 5: strongly agree). In addition, this section contained a nonordinal option of ‘not applicable’.

### Phase 2: Tool evaluation through pilot testing

#### Characteristics of HCPs included in the validity and reliability analysis

Out of the 121 HCPs who responded to the questionnaire, 14 respondents completed only the demographic section, and their responses were discarded. Of the remaining 107 HCPs, the sample was almost equally distributed by gender, with 52 males (49.5%) and 53 females (50.5%), and had a median age of 42 years (IQR = 36–50). The majority of participants were Indian (n = 25, 23.8%), followed by Filipino (n = 15, 14.3%), Jordanian (n = 14, 13.3%), Egyptian (n = 11, 10.5%), and Tunisian (n = 6, 5.7%). Most of the participants were nurses (n = 46, 43.4%), followed by physicians (n = 17, 16.0%), pharmacists (n = 9, 8.5%), and paramedics (n = 6, 5.7%). Seventy-six of the participants were from the HMC, twenty-eight from the PHCC, and one from the MoPH. The majority of participants had more than 20 years of experience in their professions (n = 38, 35.9%).

#### Structural validity: exploratory factor analysis

**Fig. 2:**
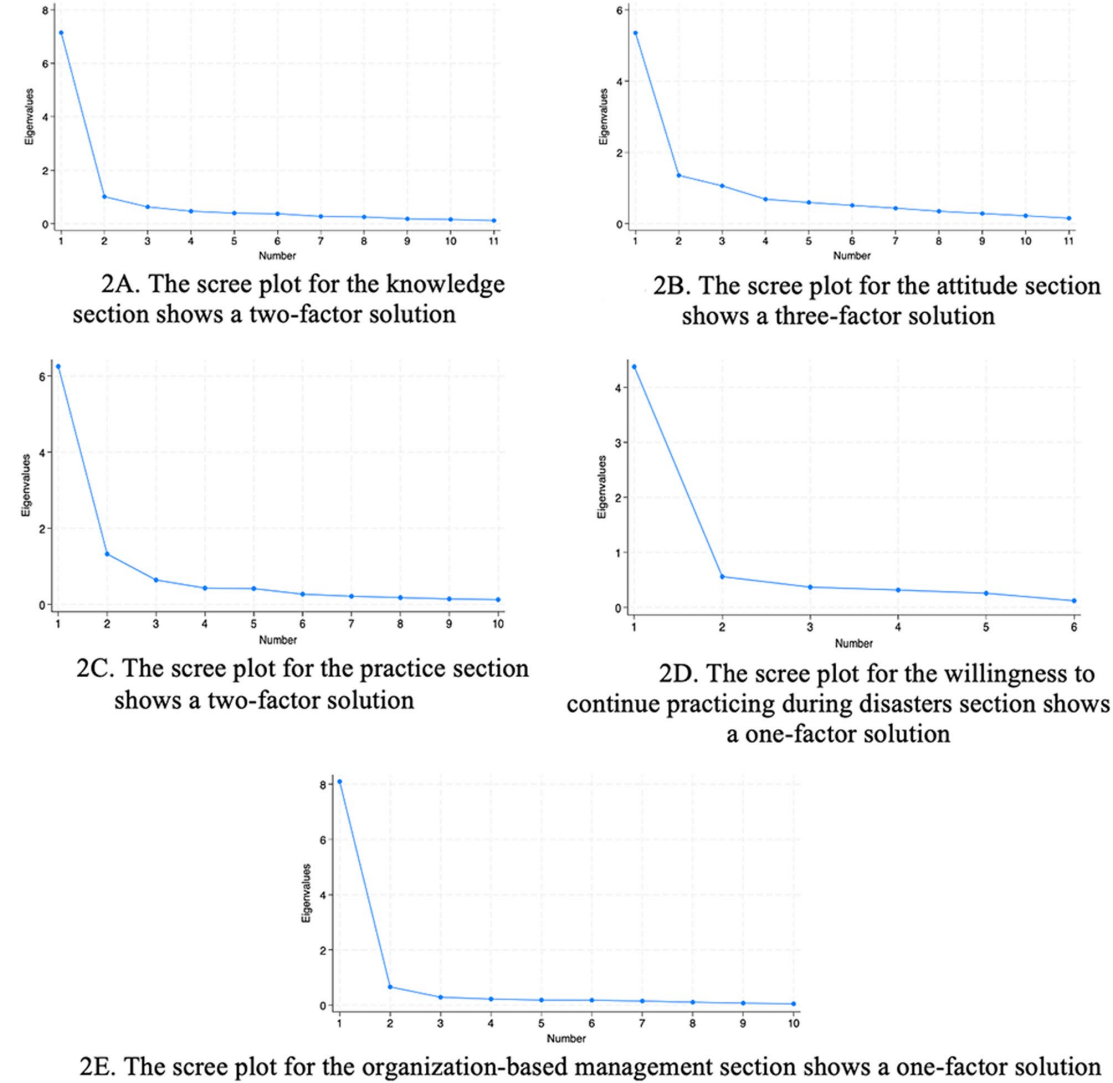
Scree plots for the five sections of Disaster Management Assessment Tool for Health Care Practitioners (DMAT_HCP)

An examination of the scree plots of each of each of the five sections suggested a two-factor solution for the knowledge section, a three-factor solution for the attitude section, a two-factor solution for the practice section, and a one-factor solution each for the willingness to practice and organization-based management section (Fig. 2).

All questionnaire items loaded significantly on their respective factors at or above 0.30 (Table 1). However, two items (i.e., ‘I know the response system for disasters in my country’ in the knowledge section, and ‘I am prepared to stay at work beyond my usual shift during disasters, if required’ in the attitude section) demonstrated cross-loadings into two or more factors and hence, were deleted.

**Table 1:** Factor loadings for exploratory factor analysis of each section of Disaster Management Assessment Tool for Health Care Practitioners (DMAT_HCP)

	Exploratory factor analysis factor loading								
	Knowledge		Attitude			Practice		Willingness to continue practicing duties	Organization-Based Management
	Familiarity with disaster response systems/interventions	knowledge of information and actions related to disaster management	Self-efficacy and self-awareness	Perceived need for a disaster plan and system	Factors influencing disaster readiness among HCPs	Actions related to knowledge and process management	Responsibilities toward self at different stages of the disaster management		
**Knowledge**									
I know the chain of command (who you should communicate with) during disasters	0.7256								
I know the response system for disasters in my organization	0.6267								
I know how to prioritize patient care in different situations during disasters	0.7792								
I know that disaster management involves multiple national organizations (e.g., hospitals, civil defense, ministry of interior, etc.)	0.7240								
I know how to deliver the basic first aid measures in a large-scale disaster event	0.6932								
I know the scope of my role in post-disaster situations	0.7736								
I know the circumstances that require immediate reporting to the responsible health agencies		0.7172							
I know how to run a disaster awareness campaign		0.7542							
I know how to collaborate with other healthcare professionals on mitigation initiatives (actions that are taken to prevent or reduce the cause, impact, and consequences of disasters)		0.7763							
I know my scope of role in managing and responding to disasters		0.6131							
I know how to find information related to disaster preparedness in my organization		0.6267							
**Attitude**									
I am interested in attending professional development programs about disaster preparedness that relate specifically to this country			0.6433						
I am prepared for rapid escalation of services (e.g., quick decision-making, resources management, task distributions … etc.)			0.6654						
I believe that my role during disasters is essential			0.5036						
I feel confident in implementing disaster management plans and procedures in my setting			0.7047						
I am able to access disaster management resources using mobile technologies (e.g., online databases)			0.6614						
I am able to perform my role in post-disaster situations			0.8453						
I believe that it is necessary to have an organizational disaster plan				0.7749					
I believe that it is necessary to have a national disaster plan				0.8173					
I believe that realistic on-scene training (at the site of incidents) is a vital component of a disaster plan					0.6882				
I feel confident in doing my job independently without the supervision of others during disasters					0.6001				
I believe that media resources (e.g., internet, TV, radio, newspapers, social media) can help my readiness to practice during disasters					0.3369				
**Practice**									
I participate in improving the current disaster plan						0.8103			
I participate in disaster drills at my workplace on a regular basis						0.5632			
I participate in the professional development programs about disaster management						0.8369			
I participate in communicating important information immediately to appropriate authorities during disasters						0.7002			
I participate in managing and supervising volunteers during disasters						0.6488			
I participate in providing defensible solutions to ethical dilemmas (e.g., resource allocation, information sharing, confidentiality) arising during disasters						0.7002			
I participate in maintaining my personal safety and the safety of others during disasters							0.6390		
I participate in responding to disasters within the scope of my professional role							0.8548		
I participate in reflecting on my own performance post-disaster							0.8397		
I participate in helping my organization to maintain functional post-disaster							0.8743		
**Willingness to continue practicing duties**									
I am willing to continue practicing my duties during a disaster even if there was no preventive measure taken by my organization to prevent it								0.8818	
I am willing to continue practicing my duties during a disaster even in the lack of adequate training about disaster management								0.9325	
I am willing to continue practicing my duties during a disaster even if I am not mentally prepared								0.7930	
I am willing to continue practicing my duties during a disaster even if I am asked to perform additional hours of duties								0.6797	
I am willing to continue practicing my duties during a disaster even if the special equipment (e.g., personal protective equipment) that are required for disaster management is unavailable								0.7854	
I am willing to continue practicing my duties in post-disaster situations even if there are no after-action reports (a report that analyzes the response to a disaster by identifying strengths to be maintained and built upon, and identifying potential areas of improvement)								0.8485	
**Organization-Based Management**									
My organization has a disaster plan									0.8242
My organization periodically updates the disaster plan									0.9178
My organization has a public health surveillance system (an ongoing, systematic collection, analysis, and interpretation of health-related data)									0.8756
My organization has all the needed medical equipment that are needed to manage an increase in the numbers of patients									0.8963
My organization makes plans for staff needs, including supplies of food, water, rest areas, and hygiene items									0.8322
My organization conducts regular disaster drills									0.9274
My organization supports the continuous professional development of healthcare professionals on topics related to disaster management									0.9264
My organization maintains a safe work environment during disasters									0.9202
My organization communicates information with the healthcare professionals throughout a disaster situation in an effective and efficient manner									0.9170
My organization recognizes the efforts of the healthcare professionals who contributed to disaster management (e.g., professional or financial recognition, etc.)									0.8378

The two factors of the knowledge section were named: 1) familiarity with disaster response systems/interventions, and 2) knowledge of information and actions related to disaster management. The loadings for the eleven knowledge items ranged from 0.61 to 0.78, and they accounted for 68.2% of the variance. For the attitude section, the three factors were named: 1) self-efficacy and self-awareness, 2) perceived need for a disaster plan and system, and 3) factors influencing disaster readiness among HCPs. The loadings for the eleven attitude items ranged from 0.34 to 0.85 and they accounted for 60.6% of the variance. Moreover, the two factors of the practice section were named: 1) actions related to knowledge and process management, and 2) responsibilities toward self at different stages of disaster management. The loadings for the ten practice items ranged from 0.60 to 0.89, and accounted for 70.2% of the variance. For the willingness to continue practicing duties, the loadings for the six items ranged from 0.68 to 0.93 and accounted for 67.9% of the variance. Whereas the loadings for the ten items of the organization-based management ranged from 0.82 to 0.93 and accounted for 79.8% of the variance.

**Fig. 3:**
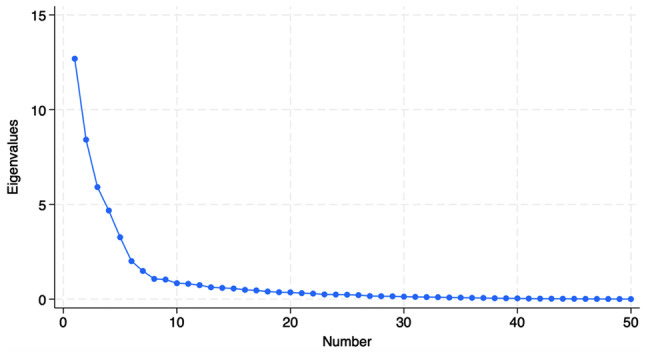
Scree plot of the entire set of Disaster Management Assessment Tool for Health Care Practitioners (DMAT_HCP) items

The examination of the Eigenvalue and the scree plot of the entire set of DMAT_HCP items (50 items) suggested initially a nine-factor solution (Fig. 3). All questionnaire items loaded significantly on their respective factors at or above 0.40 (loadings range from 0.40 to 0.93). However, eleven items demonstrated cross-loadings into two or more factors, and eight of them were deleted. This resulted in a final version of DMAT_HCP with 42 items in a six-factor solution (i.e., knowledge, attitude [perceived need for a disaster plan and system, and self-efficacy and self-awareness], practice, willingness to continue practicing duties, and organization-based management). This factor solution accounted for 77.9% of the variance, which indicated that the identified factors collectively capture a significant proportion of the variability in the data. Table 2 shows the factor loadings for EFA of DMAT_HCP entire items.

**Table 2:** Factor loadings for exploratory factor analysis of Disaster Management Assessment Tool for Health Care Practitioners (DMAT_HCP) entire items

Exploratory factor analysis factor loading						
	Knowledge	Attitude		Practice	Willingness to continue practicing duties	Organization-Based Management
		Perceived need for a disaster plan and system	Self-efficacy and self-awareness			
**Knowledge**						
I know the chain of command (who you should communicate with) during disasters	0.8013					
I know the response system for disasters in my organization	0.8358					
I know how to deliver the basic first aid measures in a large-scale disaster event	0.6569					
I know the scope of my role in post-disaster situations	0.7456					
I know the circumstances that require immediate reporting to the responsible health agencies	0.6363					
I know how to run a disaster awareness campaign	0.7546					
I know how to collaborate with other healthcare professionals on mitigation initiatives (actions that are taken to prevent or reduce the cause, impact, and consequences of disasters)	0.8084					
I know my scope of role in managing and responding to disasters	0.8392					
I know how to find information related to disaster preparedness in my organization	0.8697					
**Attitude**						
I believe that it is necessary to have an organizational disaster plan		0.7944				
I believe that it is necessary to have a national disaster plan		0.7217				
I am interested in attending professional development programs about disaster preparedness that relate specifically to this country			0.6901			
I am prepared for rapid escalation of services (e.g., quick decision-making, resources management, task distributions … etc.)			0.8294			
I believe that my role during disasters is essential			0.5687			
I feel confident in implementing disaster management plans and procedures in my setting			0.7550			
I am able to access disaster management resources using mobile technologies (e.g., online databases)			0.7156			
I am able to perform my role in post-disaster situations			0.7939			
I feel confident in doing my job independently without the supervision of others during disasters			0.5410			
I believe that media resources (e.g., internet, TV, radio, newspapers, social media) can help my readiness to practice during disasters			0.5061			
**Practice**						
I participate in communicating important information immediately to appropriate authorities during disasters				0.7576		
I participate in managing and supervising volunteers during disasters				0.7310		
I participate in providing defensible solutions to ethical dilemmas (e.g., resource allocation, information sharing, confidentiality) arising during disasters				0.8229		
I participate in maintaining my personal safety and the safety of others during disasters				0.7654		
I participate in responding to disasters within the scope of my professional role				0.8541		
I participate in reflecting on my own performance post-disaster				0.8555		
I participate in helping my organization to maintain functional post-disaster				0.8574		
**Willingness to continue practicing duties**						
I am willing to continue practicing my duties during a disaster even if there was no preventive measure taken by my organization to prevent it					0.8776	
I am willing to continue practicing my duties during a disaster even in the lack of adequate training about disaster management					0.9330	
I am willing to continue practicing my duties during a disaster even if I am not mentally prepared					0.7727	
I am willing to continue practicing my duties during a disaster even if I am asked to perform additional hours of duties					0.8098	
I am willing to continue practicing my duties during a disaster even if the special equipment (e.g., personal protective equipment) that are required for disaster management is unavailable					0.8114	
I am willing to continue practicing my duties in post-disaster situations even if there are no after-action reports (a report that analyzes the response to a disaster by identifying strengths to be maintained and built upon, and identifying potential areas of improvement)					0.8345	
**Organization-Based Management**						
My organization has a disaster plan						0.7701
My organization periodically updates the disaster plan						0.9097
My organization has a public health surveillance system (an ongoing, systematic collection, analysis, and interpretation of health-related data)						0.8563
My organization has all the needed medical equipment that are needed to manage an increase in the numbers of patients						0.8817
My organization makes plans for staff needs, including supplies of food, water, rest areas, and hygiene items						0.8656
My organization conducts regular disaster drills						0.9054
My organization supports the continuous professional development of healthcare professionals on topics related to disaster management						0.9042
My organization maintains a safe work environment during disasters						0.9306
My organization communicates information with the healthcare professionals throughout a disaster situation in an effective and efficient manner						0.8954
My organization recognizes the efforts of the healthcare professionals who contributed to disaster management (e.g., professional or financial recognition, etc.)						0.8020

#### Construct validity: convergent and divergent analyses

All DMAT_HCP items had a correlation coefficient with the score of their own section greater than 0.4. Similarly, all items had a correlation coefficient with the score of their own section greater than those computed with the scores of other sections. These findings supported the convergent and divergent validities of DMAT_HCP, indicating that the tool effectively measures the intended constructs while also demonstrating its ability to distinguish between unrelated constructs. The matrices of correlations between DMAT_HCP items of the two times of analyses are displayed in Tables 3 and 4.

**Table 3:** The matrix of correlations between Disaster Management Assessment Tool for Health Care Practitioners (DMAT_HCP) Items (first analysis)

	Kowledgen	Attitude	Practice	Willingness to continue practicing duties	Organization-Based Management
Knowledge item 1	0.661	0.366	0.224	0.194	−0.104
Knowledge item 2	0.667	0.312	0.256	0.214	−0.070
Knowledge item 3	0.786	0.318	0.259	0.255	−0.115
Knowledge item 4	0.813	0.323	0.272	0.113	−0.150
Knowledge item 5	0.844	0.281	0.224	0.150	−0.167
Knowledge item 6	0.805	0.237	0.250	−0.088	−0.136
Knowledge item 7	0.808	0.330	0.255	0.019	−0.103
Knowledge item 8	0.795	0.304	0.266	0.042	−0.181
Knowledge item 9	0.670	0.197	0.324	0.164	−0.134
Knowledge item 10	0.708	0.210	0.306	0.077	−0.084
Knowledge item 11	0.783	0.292	0.390	0.102	−0.061
Attitude item 1	0.356	0.471	0.012	0.005	−0.098
Attitude item 2	0.275	0.633	0.014	−0.030	−0.068
Attitude item 3	0.150	0.540	−0.061	0.000	−0.107
Attitude item 4	0.289	0.466	0.020	0.030	−0.173
Attitude item 5	0.237	0.637	0.000	0.223	−0.101
Attitude item 6	0.094	0.521	0.006	−0.052	−0.071
Attitude item 7	0.349	0.747	0.209	0.104	−0.096
Attitude item 8	0.299	0.651	0.028	0.091	−0.096
Attitude item 9	0.347	0.756	0.209	0.140	−0.107
Attitude item 10	0.172	0.580	0.197	0.261	−0.091
Attitude item 11	0.330	0.720	0.160	0.202	−0.046
Practice item 1	0.161	0.004	0.634	0.136	0.029
Practice item 2	0.351	0.151	0.533	0.132	0.135
Practice item 3	0.221	0.093	0.669	0.132	0.300
Practice item 4	0.326	0.138	0.805	0.103	0.078
Practice item 5	0.307	0.014	0.711	−0.004	0.078
Practice item 6	0.272	0.079	0.716	0.153	0.120
Practice item 7	0.276	0.065	0.773	0.083	0.068
Practice item 8	0.255	0.044	0.707	0.128	0.109
Practice item 9	0.149	0.085	0.748	0.158	0.082
Practice item 10	0.198	0.003	0.734	0.192	0.117
Willingness item 1	0.127	0.124	0.118	0.835	0.301
Willingness item 2	0.094	0.122	0.070	0.880	0.256
Willingness item 3	0.100	0.108	0.091	0.763	0.166
Willingness item 4	0.301	0.270	0.229	0.651	0.004
Willingness item 5	0.099	0.122	0.136	0.756	0.122
Willingness item 6	0.168	0.157	0.219	0.815	0.251
Organization item 1	−0.131	−0.102	0.061	0.117	0.828
Organization item 2	−0.136	−0.068	0.101	0.171	0.903
Organization item 3	−0.159	−0.068	0.145	0.168	0.862
Organization item 4	−0.161	−0.047	0.178	0.204	0.878
Organization item 5	−0.080	−0.138	0.157	0.122	0.645
Organization item 6	−0.080	−0.080	0.163	0.220	0.916
Organization item 7	−0.125	−0.104	0.106	0.228	0.897
Organization item 8	−0.098	−0.071	0.101	0.181	0.853
Organization item 9	−0.099	−0.028	0.135	0.307	0.902
Organization item 10	−0.142	−0.136	0.095	0.187	0.769

**Table 4:** The matrix of correlations between Disaster Management Assessment Tool for Health Care Practitioners (DMAT_HCP) Items (second analysis)

	Knowledge	Attitude	Practice	Willingness to continue practicing duties	Organization-Based Management
Knowledge item 1	0.682	0.363	0.250	0.194	−0.047
Knowledge item 2	0.679	0.318	0.199	0.214	−0.017
Knowledge item 3	0.811	0.333	0.300	0.255	−0.051
Knowledge item 4	0.818	0.330	0.249	0.113	−0.084
Knowledge item 5	0.842	0.282	0.304	0.150	−0.111
Knowledge item 6	0.779	0.246	0.211	−0.088	−0.087
Knowledge item 7	0.811	0.326	0.233	0.019	−0.054
Knowledge item 8	0.670	0.227	0.308	0.077	0.003
Knowledge item 9	0.759	0.305	0.407	0.102	0.018
Attitude item 1	0.365	0.452	0.001	0.005	−0.066
Attitude item 2	0.272	0.608	−0.005	−0.030	−0.037
Attitude item 3	0.304	0.433	0.020	0.030	−0.120
Attitude item 4	0.246	0.655	0.060	0.223	−0.075
Attitude item 5	0.087	0.515	−0.013	−0.052	−0.088
Attitude item 6	0.360	0.750	0.173	0.104	−0.104
Attitude item 7	0.290	0.650	−0.050	0.091	−0.044
Attitude item 8	0.358	0.763	0.222	0.140	−0.087
Attitude item 9	0.161	0.580	0.243	0.261	−0.049
Attitude item 10	0.322	0.752	0.187	0.202	−0.031
Practice item 1	0.325	0.149	0.762	0.054	−0.045
Practice item 2	0.321	0.074	0.758	0.026	0.066
Practice item 3	0.271	0.111	0.745	0.133	0.120
Practice item 4	0.355	0.121	0.836	0.126	0.072
Practice item 5	0.304	0.130	0.807	0.233	0.018
Practice item 6	0.201	0.155	0.801	0.194	0.026
Practice item 7	0.209	0.082	0.790	0.235	0.044
Willingness item 1	0.124	0.139	0.238	0.835	0.297
Willingness item 2	0.089	0.130	0.135	0.880	0.247
Willingness item 3	0.106	0.113	0.172	0.763	0.169
Willingness item 4	0.299	0.280	0.198	0.651	0.063
Willingness item 5	0.106	0.138	0.137	0.756	0.192
Willingness item 6	0.165	0.164	0.257	0.815	0.267
Organization item 1	−0.049	−0.055	−0.032	0.125	0.808
Organization item 2	−0.051	−0.025	0.021	0.185	0.900
Organization item 3	−0.075	−0.022	0.081	0.181	0.860
Organization item 4	−0.087	0.002	0.098	0.220	0.883
Organization item 5	−0.069	−0.061	0.066	0.180	0.824
Organization item 6	0.006	−0.041	0.083	0.236	0.912
Organization item 7	−0.043	−0.063	−0.031	0.243	0.911
Organization item 8	−0.019	−0.027	−0.025	0.235	0.909
Organization item 9	−0.031	0.030	0.056	0.333	0.904
Organization item 10	−0.079	−0.092	−0.094	0.190	0.830

#### Internal consistency analysis

All DMAT_HCP sections passed the reliability criteria (Cronbach’s alpha > 0.7). The Cronbach’s alpha scores for the knowledge, practice, willingness to continue practicing duties, and organization-based disaster management sections were 0.94, 0.92, 0.93, and 0.97, respectively, which indicated excellent internal consistency. The Cronbach’s alpha score for the attitude section was 0.89 which indicated a good internal consistency reliability. The Cronbach’s alpha score for the entire set of DMAT_HCP items was 0.90, which suggested an excellent internal consistency reliability.

### DMAT_HCP: the refined version

The final version of DMAT_HCP comprised of 42 items after removing the eight items that demonstrated cross-loadings into two or more factors. The five-point Likert scale structure and the six-factor solution (i.e., A) knowledge [9 items], B) attitude: the perceived need for a disaster plan and system, and self-efficacy and self-awareness [10 items], C) practice [7 items], D) willingness to continue practicing duties [6 items], and E) organization-based disaster management [10 items]) were retained after conducting the psychometric properties evaluation. Non-ordinal response options (i.e., not sure and not applicable) were also retained for the practice and organization-based disaster management sections, respectively.

## Discussion

This study described the development and evaluation of DMAT_HCP, an instrument designed for assessing the perceptions of HCPs regarding disaster management. DMAT_HCP was unique from other tools in the literature for several reasons.

First, a notable feature of the DMAT_HCP is its alignment with the comprehensive stages of the Disaster Management Framework (i.e., mitigation, preparedness, response, and recovery) [8]. This design feature addresses a significant gap in the field, where standardized competencies for disaster management among HCPs are lacking, and existing competency sets have not achieved universal acceptance or validation [57]. Structuring the tool around this widely recognized framework, the DMAT_HCP provides a systematic and holistic approach to identifying strengths and gaps in each critical phase of disaster management, which in turn, supports targeted interventions and evidence-based initiatives.

Second, DMAT_HCP development (i.e., content and target population validation) and evaluation (i.e., EFA, convergent and divergent validities, and reliability) involved the active participation of HCPs from diverse healthcare disciplines. This approach ensured that the DMAT_HCP is not limited to a single profession but is adaptable and inclusive, incorporating a broad spectrum of healthcare practitioners such as nurses, physicians, paramedics, laboratory technologists, pharmacists, and other allied health professionals in both frontline and administrative roles. This inclusivity allows the tool to capture the unique perspectives and contributions of various professional groups, making it particularly suited for multidisciplinary disaster management scenarios. For example, the DMAT_HCP assesses knowledge of disaster protocols, confidence in implementing disaster plans, and participation in preparedness activities such as training and drills. These dimensions are relevant across diverse work environments, from the dynamic and resource-limited settings of pre-hospital care teams to the structured environments of hospital-based practitioners and administrative staff. In contrast, while the Major Emergency Preparedness in Ireland Survey (MEPie) was designed to assess the knowledge, clinical competence, and perceptions regarding emergency planning, operations, and coordination across response agencies among a sample of HCPs, including registered nurses, paramedics, medical doctors, and administrators/managers [17], it lacked rigorous psychometric validation. Advanced psychometric testing for this assessment instrument was not conducted, suggesting less adequate evidence to support the validity and reliability of the assessment instrument [17].

Third, the adaptability of the DMAT_HCP across diverse healthcare settings is another key feature, enabling its application in multiple levels of the healthcare system. To evaluate and ensure its applicability, participants were recruited from HMC, PHCC, and the MoPH, which represent key components of Qatar’s healthcare system. The DMAT_HCP is well-suited for deployment across these distinct organizational contexts. In HMC, which provides specialized care through hospitals and advanced clinical services, the tool can assess the preparedness of HCPs managing complex cases during disasters, including coordination within specialized teams and handling mass casualties. In PHCC, which delivers primary care and community-based services, the tool can evaluate preparedness at the frontline level, focusing on early disaster response, patient triage, and community health support. For MoPH, which oversees public health governance and national healthcare strategies, the tool can be used to assess strategic preparedness, such as policy readiness, resource allocation, and inter-agency coordination during disaster scenarios. This approach highlighted the DMAT_HCP’s versatility in addressing disaster management needs at operational and policy levels which can provide actionable insights to enhance preparedness across diverse healthcare environments.

Fourth, DMAT_HCP was developed through the integration of existing questionnaires that have been used to assess disaster management across different disaster types, which enhances its applicability to a wide range of disaster contexts. This integration enables the DMAT_HCP to be used across various disaster scenarios, from natural disasters such as earthquakes and floods to more complex events such as chemical and biological disasters. This broad applicability sets the DMAT_HCP apart from many other tools that are often limited to assessing preparedness for specific disaster types or general disasters. Several assessment tools were developed to assess HCPs preparedness for disasters in general [11–13, 16, 17, 22, 25–29, 58–61], but none of these tools combine all the necessary features to be applicable to all HCPs or to be sufficiently developed and validated to be used in a global context. For instance, tools developed in the Arab region, such as the Disaster Nursing Core Competencies Scale (DNCCS) in Saudi Arabia [12], while demonstrating strong validity, are specifically designed for nursing professionals, thereby limiting their applicability to other healthcare disciplines. In contrast, tools developed by Nofal et al. (2018) [11] and Naser & Saleem (2018) [22] in Yemen, and Al-Ziftawi et al. (2020) [16] in Qatar, which target multiple healthcare professions and share similar constructs with DMAT_HCP, are primarily validated through content validity and reliability assessments. These tools, however, lack a comprehensive validation process necessary to support their broader applicability across diverse healthcare settings and various disaster contexts. Similarly, tools developed in other regions, such as the Emergency Preparedness Information Questionnaire (EPIQ) [29] in the USA and the Nurses’ Disaster Response Competencies Assessment Questionnaire (NDRCAQ) [28] in Brazil, while valuable in their respective contexts, focus primarily on the nursing profession.

Fifth, DMAT_HCP comprehensively elucidated perceptions of HCPs about disaster management at individual and organizational levels. The differentiation between individual and organizational disaster management is crucial. At the individual level, HCPs are required to have theoretical knowledge as well as practical skills, positive attitudes, and the willingness for effective disaster response [62]. On the other side, organizational disaster management involves healthcare system policies, practices, and resources [63]. HCPs’ perceptions of their roles and the organizational support available can significantly impact the quality of the disaster response [64]. Certain levels of preparedness perceived by the HCPs about the healthcare organizations, in terms of education and training of HCPs, safety precautions, access to information, and disaster risk reduction plans, can encourage and support HCPs to report to work [65–68]. Collectively, the tool’s broad applicability allows for a comprehensive assessment of disaster management at individual, team, and organizational levels which makes it a valuable resource for identifying gaps, guiding targeted interventions, and enhancing the overall resilience of healthcare systems.

One of the most important characteristics of DMAT_HCP was its robust psychometric properties, encompassing validity and reliability. Establishing construct validity is a critical aspect of any measurement instrument [47]. This study suggested that DMAT_HCP demonstrated validity with five distinct yet converging sections which supported its construct validity. The analysis of DMAT_HCP indicated the absence of spurious correlation, a common concern in assessing discriminant validity (and, to a lesser degree, convergent validity), where two sections might be correlated due to the presence of an unspecified third construct linking them [47]. In this study, convergent validity was evaluated by analyzing the correlation between items within a section, and divergent validity was evaluated by determining if an item’s correlation with its hypothesized section was higher than its correlation with other sections. Using a similar approach, Han and Chun (2010) assessed the convergent and divergent validities for a Korean version of the disaster preparedness evaluation tool (DPET-K) for nurses after establishing the model fit with the confirmatory factor analysis [69]. Factor loading, significance, average variance extracted (AVEs) > 0.50, and construct reliability > 0.70 were examined to confirm convergent validity. Whereas, the discriminant validity was confirmed when factors had higher AVEs than the squared correlation coefficient [69]. Another common approach to evaluating convergent and divergent validities is to evaluate them across instruments rather than within an instrument [32, 33, 47]. Instruments that are meant to measure comparable constructs are expected to correlate more highly with one another than with scales meant to measure unrelated constructs [32, 33, 47]. Nevertheless, components of the validity unique to the instrument under research, such as how effectively it captures all pertinent aspects of the construct, may be overlooked by comparing it just to comparable instruments [70, 71]. Moreover, true convergent or divergent validity may be misrepresented by confounding variables introduced by variations in instrument design, target demographics, and administration techniques [72].

In this study, DMAT_HCP demonstrated adequate internal consistency examined using the reliability statistic—Cronbach’s alpha for each section. Demonstrating adequate internal reliability indicates the robustness of the validity and suggests that the items accurately represent the intended domain [47]. Moreover, the adequacy of internal consistency can anticipate the stability of DMAT_HCP over time when the instrument is administered repeatedly (i.e., test-retest reliability) because these two concepts are mathematically related [47]. It is worth mentioning that although the alpha score for the ‘Attitude’ section was the lowest compared to other sections of DMAT_HCP, it still indicated good reliability and is comparable to other studies in disaster management [16, 58].

### Limitations

The DMAT_HCP, as a self-reported instrument, is subject to potential biases, such as social desirability bias, which may limit its ability to objectively assess HCPs proficiency in disaster management. Additionally, the online administration of the tool presents specific challenges, such as its reliance on participants having stable internet access and familiarity with digital platforms. Moreover, the absence of a facilitator during completion may lead to misinterpretation of questions and reduced engagement, potentially resulting in lower response rates and incomplete data. The development of DMAT_HCP employed a deductive approach to generating items, which involved synthesizing items from a literature review and pre-existing questionnaires. However, it is recommended to complement this approach with the inductive approach for item generation of new questionnaires through conducting focus groups or individual interviews. Due to time constraints, the incorporation of an inductive approach was not feasible in this study. Moreover, while the five-point response options of the questionnaire Likert scales allowed for distinctions in respondents’ perceptions and hence yielded richer data for analysis, it might have introduced some complexity in responding to the items. In addition, while the development of DMAT_HCP was based on international pre-tested questionnaires, the evaluation of its psychometric properties was restricted to HCPs in Qatar. Furthermore, the EFA findings should be interpreted with caution as the analysis was performed on the minimal recommended sample size which might have not accurately reflected the estimates of factor loadings or the underlying structure of the variables in the broader population. Also, future studies need to run an outlier analysis to prevent the distortion of factor structures. While Cronbach’s alpha is commonly used and generally accepted, there is an ongoing debate about alternative reliability statistics (e.g., Raykov’s rho) which are believed to provide enhancements and are gaining increasing acceptance over Cronbach’s alpha [32].

### Future research and recommendations

Future improvements should also include a robust attempt to achieve more balanced and representative samples of HCPs in the item generation and testing phases. This could include running focus groups for item generation, with balanced participation across HCP categories. In addition, to enhance national preparedness, future research and readiness initiatives should prioritize inclusivity by incorporating HCPs from private and semi-private sectors. Such an approach will provide a more comprehensive understanding of preparedness at the national level and facilitate the development of strategies that engage and empower all stakeholders in disaster management. Moreover, future research is needed to further validate the psychometric properties of DMAT_HCP, including performing confirmatory factor analysis to confirm the resultant factors from the EFA, predictive validity to assess the ability of DMAT_HCP to predict HCPs’ behaviors related to disaster management, and more advanced analysis of convergent and divergent validity (e.g., multi scale-multi-method matrix). To ensure global applicability, the DMAT_HCP instrument requires evaluation in diverse international contexts beyond its initial development and validation in Qatar. This broader assessment would strengthen its validity and reliability across a wider range of cultural and healthcare settings. Moreover, DMAT_HCP can be potentially utilized to serve a dual purpose, such that it can discriminate between individuals with poor, fair, or good perceptions about disaster management (*discriminative purpose*), while also facilitating the monitoring of the changes in perceptions over time (*evaluative purpose*). However, further analyses of sensitivities and responsiveness are warranted to support the dual purpose of DMAT_HCP.

Following validation, the DMAT_HCP is recommended for use across diverse healthcare settings to assess disaster management among HCPs. Its adaptability to various healthcare disciplines and disaster contexts makes it a valuable tool for broad application. Moreover, the tool can be incorporated into disaster management training initiatives to identify gaps in knowledge and readiness among HCPs, which can guide the development of targeted, evidence-based training programs to address specific weaknesses. Additionally, regular use of the DMAT_HCP in healthcare institutions is encouraged to monitor improvements in preparedness over time. This would enable continuous evaluation of training effectiveness and preparedness strategies. The use of DMAT_HCP provides policymakers and stakeholders with actionable insights to optimize training, resource allocation, and strategic planning, thereby strengthening healthcare systems’ capacity to effectively manage disasters.

## Conclusions

The newly developed Disaster Management Assessment Tool for Health Care Practitioners (DMAT_HCP) is a self-administered tool that comprehensively assesses the perceptions of HCPs regarding their disaster management knowledge, attitude, practices, willingness to continue practicing duties during disasters, as well as their perceptions about the level of preparedness of healthcare organizations to manage disasters. Items in each section were developed based on a review of existing validated tools and were aligned with the four stages of the Disaster Management Framework. Two rounds of expert review were used to assess content validity, while HCPs evaluated face validity. The validity of DMAT_HCP was established through pilot testing with HCPs from different health disciplines, by employing multiple validity assessment tests. These tests included structural validity using exploratory factor analyses, and construct validity through the establishment of convergent and divergent validities. Factor analysis of the five sections of the DMAT_HCP suggested a two-factor solution for the knowledge section, a three-factor solution for the attitude section, a two-factor solution for the practice section, and a one-factor solution each for the willingness to practice and organization-based management section. However, the factor analysis of the entire set of DMAT_HCP items suggested a six-factor solution representing the items of knowledge, two sub-domains of attitude, practice, willingness to practice, and organization-based management. DMAT_HCP demonstrated construct validity with five distinct, yet converging sections and adequate internal consistency. The study suggested that DMAT_HCP is both conceptually and methodologically sound, demonstrating validity and reliability. DMAT_HCP offers a comprehensive, globally applicable assessment of disaster management, suitable for use across various healthcare professions, settings, disaster types, and management phases.

## Data Availability

The datasets used and/or analysed during the current study are available from the corresponding author on reasonable request.

## Abbreviations

DMAT_HCP: Disaster Management Assessment Tool for Health Care Practitioners
HCPs: Healthcare Practitioners
HMC: Hamad Medical Corporation
PHCC: Primary Health Care Corporation
MoPH: Ministry of Public Health
DPET: Disaster Preparedness Evaluation Tool
EPIQ: Emergency Preparedness Information Questionnaire
AVEs: Average variance extracted
MEPie: Major Emergency Preparedness in Ireland Survey
DNCCS: Disaster Nursing Core Competencies Scale
CVI: Content Validity Index
EFA: Exploratory Factor Analysis
KMO: Kaiser–Meyer–Olkin
BTS: Bartlett’s Test of Sphericity
